# Supplementary material for: Gelatinous filter feeders increase ecosystem efficiency
Source: Commun Biol. 2024 Aug 23;7:1039. doi: 10.1038/s42003-024-06717-1 (PMC11343865; doi:10.1038/s42003-024-06717-1)
Supplement: Supplementary file 2 — Supplemental Material [file 42003_2024_6717_MOESM2_ESM.pdf]

**Supplementary Information**

for:

# Gelatinous filter feeders increase ecosystem efficiency

Michael R. Stukel*[,c,1,2], Moira Décima*[,3], Christian K. Fender[1], Andres Gutierrez-Rodriguez[4], Karen E. Selph[5]

[1]Dept. of Earth, Ocean, and Atmospheric Science, Florida State University, Tallahassee, FL

[2]Center for Ocean-Atmospheric Prediction Studies, Florida State University, Tallahassee, FL

[3]Scripps Institution of Oceanography, University of California San Diego, SA

[4]National Institute of Water and Atmospheric Research (NIWA), Wellington, New Zealand

[5]Dept. of Oceanography, University of Hawaii at Manoa, Honolulu, HI

*These authors contributed equally to this study

[c]Corresponding author : mstukel@fsu.edu

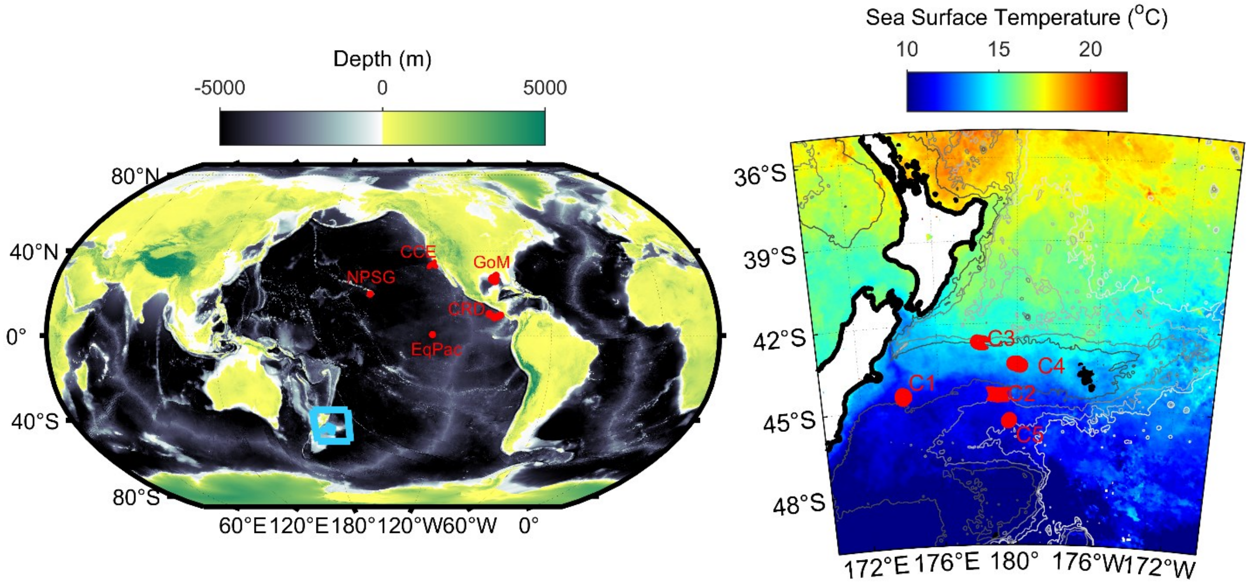


**Supp. Fig. 1** – Study locations. Left panel shows the primary study location (light blue box) as well as the locations of additional samples from other regions that were used to investigate broader scale patterns in ecosystem transfer efficiency (red dots; CCE = California Current Ecosystem, NPSG = North Pacific Subtropical Gyre, EqPac = Equatorial Pacific, CRD = Costa Rica Dome, GoM = Gulf of Mexico). Right panel shows greater detail of the study region (red dots are locations of Lagrangian Experiments, Cycles (C) 1 - 5). C1 = Subantarctic with influence of Southland Current (+Salp), C2 = Subantarctic (+Salp), C3 = Subtropical (No Salp), C4 = Subtropical (+Salp), C5 = Subantarctic (No salp). Background color is MODIS Aqua average monthly sea surface temperature for October, 2018.

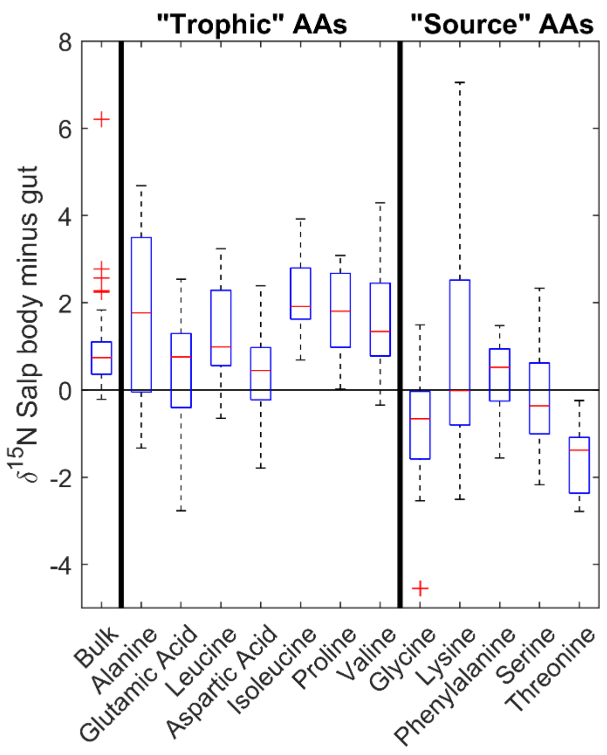


**Supp. Fig. 2** – Boxplot showing difference between $\delta^{15}N$ of salp bodies and salp guts for bulk nitrogen (left box) and amino acids (AA, all other boxes). Outliers (red + symbols) are values that are more than 1.5 times the interquartile range from either the 25 or 75$^{th}$ percentile.

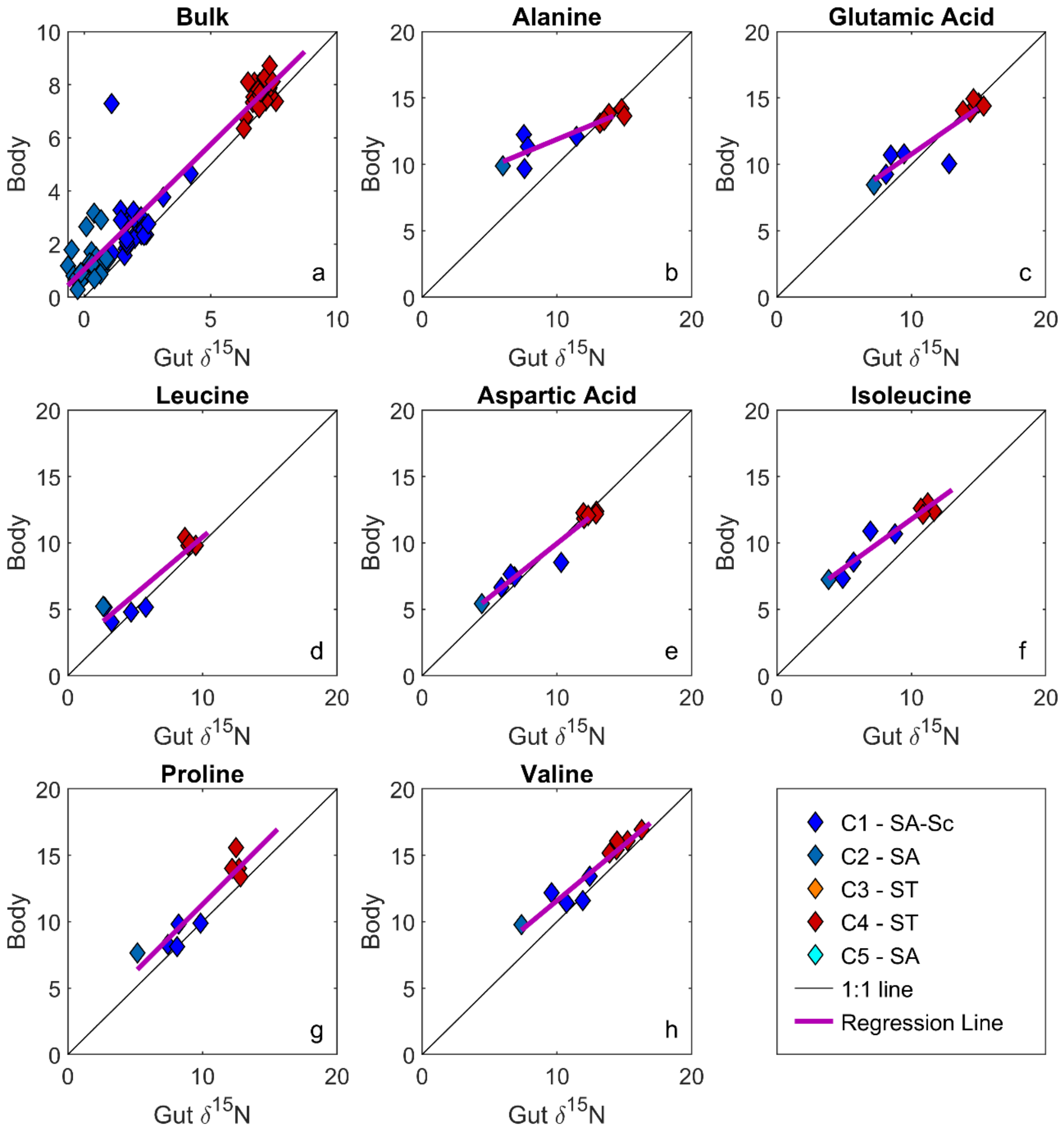


**Supp. Fig. 3** – Salp body $\delta^{15}$N plotted as a function of salp gut $\delta^{15}$N for bulk (a), alanine (b), glutamic acid (c), leucine (d), aspartic acid (e), isoleucine (f), proline (g), and valine (h). Black line is the 1:1 line. Magenta line is a linear regression. SA-Sc = Southland-Current-Influenced Subantarctic, SA = Subantarctic, ST = Subtropical.

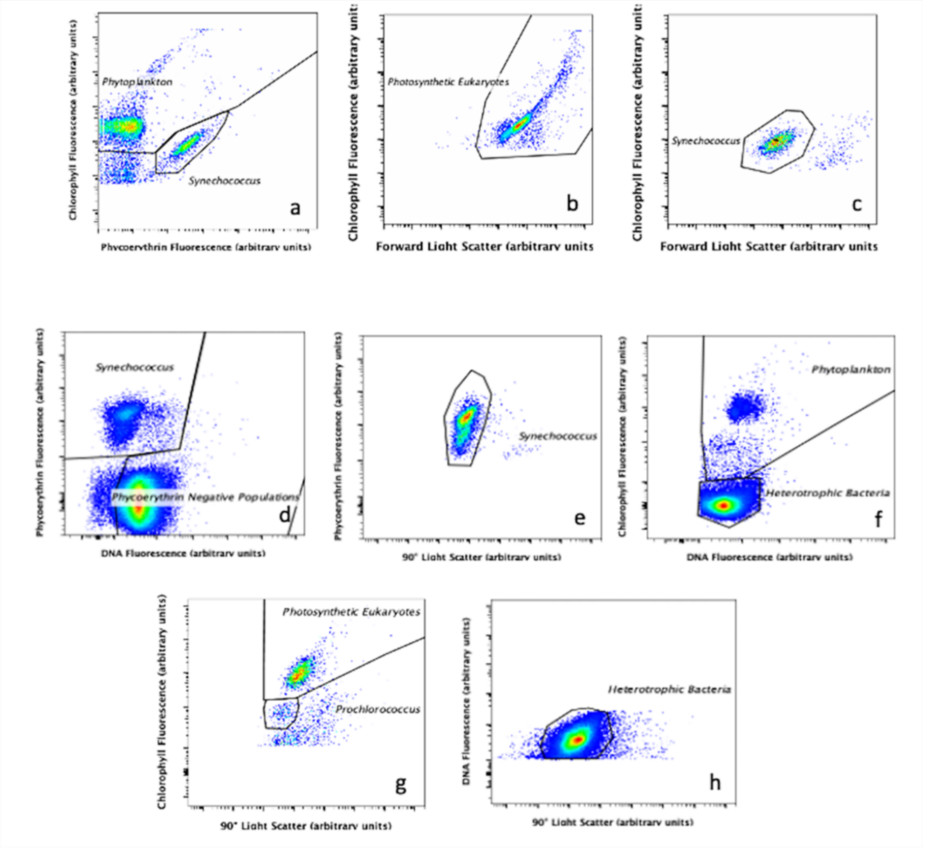


Supp. Fig. 4. Flow cytometry gating scheme for phytoplankton and heterotrophic bacteria analyzed on Accuri C6 (live, unstained, panels a-c) and CytoFLEX S (preserved, Hoechst-DNA stained, panels d-h). Accuri data: a) Chlorophyll vs. phycoerythrin fluorescence, showing gate for Synechococcus (phycoerythrin-positive) vs. all other phytoplankton. b) Phytoplankton further gated on chlorophyll vs. forward light scatter. c) Synechococcus further gated on chlorophyll vs forward light scatter. CytoFLEX data: d) Phycoerythrin-positive Synechococcus separated from all other populations using phycoerythrin vs. DNA fluorescence. e) Further refinement of Synechococcus using phycoerythrin fluorescence vs. 90° light scatter. f) Separation of phytoplankton from heterotrophic bacteria using chlorophyll vs. DNA fluorescence. g) Phyotplankton separated into photosynthetic eukaryotes and Prochlorococcus using chlorophyll vs. 90° light scatter. h) Further refinement of heterotrophic bacteria using DNA fluorescence vs. 90° light scatter.

| | | | | Mesozooplankton Grazing (mg C $m^{-2}$ $d^{-1}$) | | | | | | | Mesozooplankton Trophic Position | | | | | | |
|---|---|---|---|---|---|---|---|---|---|---|---|---|---|---|---|---|
| Cruise | Cycle | NPP (mg C $m^{-2}$ $d^{-1}$) | Protistan Grazing (mg C $m^{-2}$ $d^{-1}$) | 0.2 - 0.5 | 0.5 - 1 | 1 - 2 | 2 - 5 | >5 | Phototrophic Flagellate Trophic Position | Heterotrophic Protist Trophic Position | 0.2 - 0.5 | 0.5 - 1 | 1 - 2 | 2 - 5 | >5 | Gelatinous filter feeder grazing (mg C $m^{-2}$ $d^{-1}$) | Gelatinous filter feeder mean size (mm) |
| CRD | 2 | 1200 [a] | 1152 [a] | 130.45 [b] | 115.67 [b] | 94.83 [b] | 29.05 [b] | 7.99 [b] | | | 2.18 [c] | 2.17 [c] | 2.73 [c] | 2.62 [c] | 2.62 [c] | 123.43 [d] | 98.47 [d] |
| CRD | 3 | 1008 [a] | 648 [a] | 191.45 [b] | 210.61 [b] | 264.85 [b] | 94.36 [b] | 10.33 [b] | 1.22 [a] | 2.28 [a] | 2.39 [a] | 2.35 [a] | 2.56 [a] | 2.71 [a] | 2.56 [a] | 23.38 [d] | 38.55 [d] |
| CRD | 4 | 888 [a] | 408 [a] | 75.35 [b] | 70.41 [b] | 75.59 [b] | 22.63 [b] | 0.82 [b] | | | 2.13 [c] | 2.30 [c] | 2.52 [c] | 2.56 [c] | 2.56 [c] | 196.51 [d] | 176.93 [d] |
| CRD | 5 | 1104 [a] | 456 [a] | 213.77 [b] | 192.54 [b] | 156.11 [b] | 77.26 [b] | 7.11 [b] | 1.12 [a] | 2.21 [a] | 2.32 [a] | 2.34 [a] | 2.68 [a] | 2.75 [a] | 2.53 [a] | 95.71 [d] | 160.13 [d] |
| Equatorial Biocomplexity | | 672 [e] | 602 [e] | 57.31 [f] | 63.63 [f] | 56.17 [f] | 30.65 [f] | 9.24 [f] | | | 2.58 [c] | 2.41 [c] | 2.15 [c] | 2.14 [c] | 2.14 [c] | 0.00 | |
| Cyclone Opal | Out | 552 [g] | 325 [g] | 19.48 [h] | 16.12 [h] | 10.89 [h] | 2.04 [h] | 0.15 [h] | | | 2.35 [c] | 2.49 [c] | 2.66 [c] | 2.65 [c] | 2.65 [c] | 0.00 | |
| CCEP0605 | 1 | 4184 [i] | 1916 [j] | 553.18 [k] | 392.18 [k] | 333.81 [k] | 59.29 [k] | 10.22 [k] | 1.00 [l] | 2.37 [l] | 2.65 [l] | 2.65 [l] | 2.65 [l] | 2.65 [l] | 2.65 [l] | 0.00 | |
| CCEP0605 | 2 | 563 [i] | 436 [j] | 101.19 [k] | 80.68 [k] | 14.34 [k] | 4.30 [k] | 1.32 [k] | 1.00 [l] | 2.36 [l] | 2.75 [l] | 2.75 [l] | 2.75 [l] | 2.75 [l] | 2.75 [l] | 0.00 | |
| CCEP0605 | 3 | 4382 [i] | 3156 [j] | 1494.49 [k] | 363.89 [k] | 122.55 [k] | 36.49 [k] | 2.26 [k] | 1.00 [l] | 2.33 [l] | 2.63 [l] | 2.63 [l] | 2.63 [l] | 2.63 [l] | 2.63 [l] | 0.00 | |
| CCEP0605 | 4 | 1474 [i] | 656 [j] | 114.08 [k] | 121.22 [k] | 38.56 [k] | 2.87 [k] | 0.17 [k] | 1.00 [l] | 2.33 [l] | 2.68 [l] | 2.68 [l] | 2.68 [l] | 2.68 [l] | 2.68 [l] | 0.00 | |
| CCEP0605 | 5 | 483 [i] | 450 [j] | 37.49 [k] | 33.78 [k] | 10.51 [k] | 2.88 [k] | 1.36 [k] | 1.00 [l] | 2.28 [l] | 2.88 [l] | 2.88 [l] | 2.88 [l] | 2.88 [l] | 2.88 [l] | 0.00 | |
| CCEP0704 | 1 | 1233 [i] | 661 [j] | 1134.41 [k] | 664.72 [k] | 143.75 [k] | 30.51 [k] | 25.50 [k] | 1.00 [m] | 2.35 [m] | 2.44 [m] | 2.39 [m] | 2.41 [m] | 2.37 [m] | 2.38 [m] | 0.00 | |
| CCEP0704 | 2 | 587 [i] | 716 [j] | 88.46 [k] | 52.90 [k] | 44.19 [k] | 3.29 [k] | 1.07 [k] | 1.00 [m] | 2.15 [m] | 2.36 [m] | 2.34 [m] | 2.78 [m] | 2.81 [m] | 2.81 [m] | 0.00 | |
| CCEP0704 | 4 | 2314 [i] | 1283 [j] | 1025.31 [k] | 459.84 [k] | 216.11 [k] | 54.62 [k] | 22.25 [k] | 1.00 [m] | 2.19 [m] | 2.65 [m] | 2.54 [m] | 2.88 [m] | 2.87 [m] | 2.82 [m] | 0.00 | |
| CCEP0810 | 1 | 554 [i] | 472 [n] | 37.92 [o] | 40.03 [o] | 22.09 [o] | 6.53 [o] | 1.19 [o] | 1.00 [m] | 2.57 [m] | 2.60 [m] | 2.56 [m] | 3.13 [m] | 3.11 [m] | 3.12 [m] | 0.00 | |
| CCEP0810 | 2 | 484 [i] | 243 [n] | 105.54 [o] | 93.22 [o] | 28.53 [o] | 6.23 [o] | 1.06 [o] | 1.00 [m] | 2.35 [m] | 2.62 [m] | 2.53 [m] | 3.15 [m] | 3.12 [m] | 3.12 [m] | 0.00 | |
| CCEP0810 | 3 | 893 [i] | 357 [n] | 189.12 [o] | 277.92 [o] | 89.85 [o] | 5.36 [o] | 2.36 [o] | 1.00 [m] | 2.37 [m] | 2.33 [m] | 2.32 [m] | 3.07 [m] | 2.88 [m] | 2.88 [m] | 0.00 | |
| NF17 | 1 | 308 [p] | 524 [q] | 9.90 [r] | 11.24 [r] | 7.58 [r] | 4.74 [r] | 0.00 [r] | 1.58 [s] | 2.53 [s] | 3.33 [s] | 3.57 [s] | 3.57 [s] | 3.97 [s] | 3.97 [s] | 0.00 | |
| NF18 | 5 | 351 [p] | 420 [q] | 14.19 [r] | 14.42 [r] | 15.36 [r] | 8.12 [r] | 1.01 [r] | 1.70 [s] | 2.44 [s] | 3.21 [s] | 3.36 [s] | 3.36 [s] | 3.80 [s] | 3.80 [s] | 0.00 | |

**Supplementary Table 1** – Food-web data from other ecosystems. [a1, b2, c]Trophic Position estimates for >5-mm zooplankton were not available and we assumed they were equal to trophic position of 2-5 mm zooplankton. This foodweb model included mixotrophic flagellates, which were the main phototrophic prey of mesozooplankton, hence their trophic position is included in calculations [3], [d4], [e]Regional Average [5], [f]Regional Average [6], [g]Results from outside eddy conditions were used, because they seemed most comparable to conditions sampled on HOT cruises for trophic position [7], [h8], [i9], [j]data from [10] converted to carbon units following [11] [k]data from [12] converted to carbon units following [11], [l]Trophic position was only available for bulk (not size-fractionated) mesozooplankton [11], [m]Trophic positions were available for <1-mm epipelagic resident, <1-mm vertically migrating, >1-mm epipelagic resident, and >1-mm vertically migrating zooplankton. These were converted to size classes by weighting to the proportion of vertical migrators in each size class from [13]. [n]data from [10] converted to carbon units following [14]. [o]data from [12] converted to carbon units following [14]. [p15], [q 16], [r]data from [17] converted to carbon units following [16], [s]Trophic position was available for taxon-specific groups and was converted to size classes assuming that herbivorous non-vertically migrating zooplankton could be in all size classes, appendicularians were <0.5 mm, cladocerans were 0.5 – 1 mm, non-vertically migrating calanoid copepods were 0.5 – 2 mm, , herbivorous vertically migrating zooplankton were >1 mm, and vertically migrating calanoid copepods were 0.5 – 2 mm. Trophic positions were then assigned based on the biomass-weighted means of appropriately sized groups. This foodweb model included mixotrophic flagellates, which were the main phototrophic prey of mesozooplankton, hence their trophic position is included in calculations [16].

| | Bulk | Alanine | Glutamic Acid | Leucine | Aspartic Acid | Isoleucine | Proline | Valine |
|---|---|---|---|---|---|---|---|---|
| Mean | 0.87 | 1.70 | 0.52 | 1.31 | 0.42 | 2.24 | 1.77 | 1.64 |
| St. Err. | 0.10 | 0.47 | 0.33 | 0.28 | 0.27 | 0.24 | 0.27 | 0.31 |

**Supplementary Table 2 -** Trophic discrimination factors (TDFs) for bulk $\delta^{15}N$ and trophic amino acids as calculated based on the difference between salp body tissue and gut contents.

## References


1 Stukel, M. R., Décima, M., Landry, M. R. & Selph, K. E. Nitrogen and isotope flows through the Costa Rica Dome upwelling ecosystem: The crucial mesozooplankton role in export flux. *Global Biogeochem. Cycles* **32**, 1815-1832 (2018). https://doi.org:10.1029/2018GB005968

2 Décima, M., Landry, M. R., Stukel, M. R., Lopez-Lopez, L. & Krause, J. W. Mesozooplankton biomass and grazing in the Costa Rica Dome: amplifying variability through the plankton food web. *J. Plankton Res.* **38**, 317-330 (2016). https://doi.org:10.1093/plankt/fbv091

3 Décima, M. Zooplankton trophic structure and ecosystem productivity. *Mar. Ecol. Prog. Ser.* **692**, 23-42 (2022).

4 Décima, M., Stukel, M. R., López-López, L. & Landry, M. R. The unique ecological role of pyrosomes in the Eastern Tropical Pacific. *Limnol. Oceanogr.* **64**, 728-743 (2019). https://doi.org:10.1002/lno.11071

5 Stukel, M. R. & Landry, M. R. Contribution of picophytoplankton to carbon export in the equatorial Pacific: A re-assessment of food-web flux inferences from inverse models. *Limnol. Oceanogr.* **55**, 2669-2685 (2010). https://doi.org:10.4319/lo.2010.55.6.2669

6 Décima, M., Landry, M. R. & Rykaczewski, R. R. Broad-scale patterns in mesozooplankton biomass and grazing in the eastern equatorial Pacific. *Deep-Sea Res. II* (2011).

7 Benitez-Nelson, C. R. *et al.* Mesoscale eddies drive increased silica export in the subtropical Pacific Ocean. *Science* **316**, 1017-1021 (2007).

8 Landry, M. R., Decima, M., Simmons, M. P., Hannides, C. C. S. & Daniels, E. Mesozooplankton biomass and grazing responses to Cyclone Opal, a subtropical mesoscale eddy. *Deep-Sea Res. II* **55**, 1378-1388 (2008). https://doi.org:https://doi.org/10.1016/j.dsr2.2008.01.005

9 Goericke, C. C. E. L. a. R. (ed Environmental Data Initiative) (2023).

10 Landry, C. C. E. a. M. (ed Environmental Data Initiative) (2021).

11 Stukel, M. R. *et al.* Do inverse ecosystem models accurately reconstruct plankton trophic flows? Comparing two solution methods using field data from the California Current. *J. Mar. Sys.* **91**, 20-33 (2012). https://doi.org:10.1016/j.jmarsys.2011.09.004

12 Ohman, C. C. E. L. a. M. Gut Fluorescence measurements of mesozooplankton grazing on autotrophic prey. Samples collected in the CCE-LTER region on Process Cruises from 2006 to the present. Summaries for each Lagrangian Cycle. ver 2. . *Environmental Data Initiative* (2022). https://doi.org:10.6073/pasta/2b0dd8abed1a6ae028e34e305e88c1af

13 Ohman, C. C. E. L. a. M. Dry weight biomass measurements of net-collected mesozooplankton. Samples collected in the CCE-LTER region on Process Cruises from 2006 to the present. Summaries for each Lagrangian Cycle. ver 2. . *Environmental Data Initiative* (2022). https://doi.org:10.6073/pasta/02f5f4cf15817da0892aef71787e8768

14 Kelly, T. B. *et al.* The Importance of Mesozooplankton Diel Vertical Migration for Supporting a Mesopelagic Ecosystem. *Front. in Mar. Sci.* **6**, 508 (2019). https://doi.org:10.3389/fmars.2019.00508

15 Yingling, N. *et al.* Taxon-specific phytoplankton growth, nutrient limitation, and light limitation in the oligotrophic Gulf of Mexico. *J. Plankton Res.* **44**, 656-676 (2022). https://doi.org:10.1093/plankt/fbab028

16 Stukel, M. R. *et al.* Plankton food webs of the Gulf of Mexico spawning grounds of Atlantic Bluefin tuna. *J. Plankton Res.* **44**, 763–781 (2022). https://doi.org:10.1093/plankt/fbab023

17 Landry, M. R. & Swalethorp, R. Mesozooplankton biomass, grazing and trophic structure in the bluefin tuna spawning area of the oceanic Gulf of Mexico. *J. Plankton Res.* (2021).
