# Supplementary material for: Gelatinous filter feeders increase ecosystem efficiency
Source: Commun Biol. 2024 Aug 23;7:1039. doi: 10.1038/s42003-024-06717-1 (PMC11343865; doi:10.1038/s42003-024-06717-1)
Supplement: Supplementary file 3 — Description of Additional Supplementary Files [file 42003_2024_6717_MOESM3_ESM.pdf]

Description of Additional Supplementary Files

File name: Supplementary Data 1
Description: Bulk stable isotopes (^{13}C and ^{15}N) for water column particulate organic matter collected on GF/F filters.

File name: Supplementary Data 2
Description: Bulk stable isotopes (^{13}C and ^{15}N) for size-fractionated mesozooplankton samples.

File name: Supplementary Data 3
Description: Bulk stable isotopes (^{13}C and ^{15}N) for salp bodies and guts.

File name: Supplementary Data 4
Description: Compound-specific isotopic analysis of amino acids for size-fractionated mesozooplankton samples.

File name: Supplementary Data 5
Description: Compound-specific isotopic analysis of amino acids for salp bodies and guts.

File name: Supplementary Data 6
Description: Zip file containing all Matlab code needed to generate figures for this manuscript.
